# Biochemical recurrence in patients with prostate cancer after primary definitive therapy: treatment based on risk stratification

**DOI:** 10.1038/s41391-023-00712-z

**Published:** 2023-09-07

**Authors:** Neal D. Shore, Judd W. Moul, Kenneth J. Pienta, Johannes Czernin, Martin T. King, Stephen J. Freedland

**Affiliations:** 1 Carolina Urologic Research Center, Myrtle Beach, SC USA; 2 Duke Cancer Institute, Duke University, Durham, NC USA; 3 Johns Hopkins School of Medicine, Baltimore, MD USA; 4 David Geffen School of Medicine, University of California, Los Angeles, Los Angeles, CA USA; 5 Brigham and Women’s Hospital and Dana-Farber Cancer Institute, Boston, MA USA; 6 Samuel Oschin Comprehensive Cancer Center, Cedars-Sinai Medical Center, Los Angeles, CA USA; 7 Veterans Affairs Medical Center, Durham, NC USA

**Keywords:** Prostate cancer, Prostate cancer

## Abstract

**Background:**

Nearly one-third of patients with prostate cancer (PCa) experience biochemical recurrence (BCR) after primary definitive treatment. BCR increases the risk of distant metastasis and mortality in patients with prognostically unfavorable features. These patients are best managed with a tailored treatment strategy incorporating risk stratification using clinicopathological factors, next-generation imaging, and genomic testing.

**Objective:**

This narrative review examines the utility of risk stratification for the management of patients with BCR in the context of clinical trial data, referencing the latest recommendations by European and US medical societies.

**Methods:**

PubMed was searched for relevant studies published through May 21 2023 on treatment of patients with BCR after radical prostatectomy (RP) or external beam radiotherapy (EBRT).

**Results:**

European and US guidelines support the risk-stratified management of BCR. Post-RP, salvage EBRT (with or without androgen deprivation therapy [ADT]) is an accepted treatment option for patients with BCR. Post-EBRT, local salvage therapies (RP, cryotherapy, high-intensity focused ultrasound, stereotactic body radiotherapy, and low-dose-rate and high-dose-rate brachytherapy) have demonstrated comparable relapse-free survival rates but differing adverse event profiles, short and long term. Local salvage therapies should be used for local-only relapses while ADT should be considered for regional or distant relapses. In practice, patients often receive ADT, with varying guidance for intermittent ADT vs. continuous ADT, due to consideration of quality-of-life effects.

**Conclusions:**

Despite a lack of consensus for BCR treatment among guideline associations and medical societies, risk stratification of patients is essential for personalized treatment approaches, as it allows for an informed selection of therapeutic strategies and estimation of adverse events. In lower-risk disease, observation is recommended while in higher-risk disease, after failed repeat local therapy, ADT and/or clinical trial enrollment may be appropriate. Results from ongoing clinical studies of patients with BCR should provide consensus for management.

## Introduction

In 2020, prostate cancer (PCa) was the second most common malignancy diagnosed in men with an incidence of 1414259 cases worldwide, representing 7.3% of all new cancers globally [1]. Older age, African American race, and a family history of PCa are established risk factors [2]. For patients with more aggressive localized disease, and when intervention is recommended, several options exist, including but not limited to radical prostatectomy (RP) and external beam radiation therapy (EBRT). Despite early intervention, 20–50% of men with PCa will develop biochemical recurrence (BCR) within 10 years after initial definitive therapy, characterized by a rising serum prostatic-specific antigen (PSA) [3, 4]. Of note, BCR can represent local recurrence and increases the risk of metastasis and PCa-specific mortality (PCSM) in patients with prognostically unfavorable pre- and post-treatment clinicopathological factors, including a high Gleason score (GS) and a short PSA doubling time (PSADT) [5–7]. Thus, patients with BCR would be best managed with a tailored treatment strategy that incorporates risk stratification using pathological grade group, PSADT, conventional imaging, molecular targeted imaging (MTI), also referred to as next-generation imaging, and possibly genomic testing [8–10]. The application of MTI in the diagnostic evaluation and management of patients with BCR will be discussed in a companion review.

Over the last decade, there has been important progress in the personalized management of BCR [11–13]. However, there is a current lack of consensus among guideline associations and medical societies regarding the most effective treatments for BCR. In the absence of this guidance, it is important for the uro-oncology community to be aware of the latest clinical evidence.

This narrative review comprehensively evaluates clinical trial data to summarize treatment approaches for BCR, including lifestyle interventions. Of note, we focused on BCR after whole gland treatment. We also discuss the extent to which clinicians apply guideline recommendations and impact of risk stratification on patient management, with reference to the latest recommendations by the American Urology Association/American Society for Radiation Oncology/Society of Urologic Oncology (AUA/ASTRO/SUO), American Society of Clinical Oncology (ASCO), European Association of Urology/European Association of Nuclear Medicine/European Society for Radiotherapy & Oncology/European Society of Urogenital Radiology/International Society of Urological Pathology/International Society of Geriatric Oncology (EAU/EANM/ESTRO/ESUR/ISUP/SIOG) and National Comprehensive Cancer Network® (NCCN®) [8, 9, 14–16].

## Methods

A comprehensive search was conducted through PubMed to identify relevant publications on treatment strategies in patients with BCR and negative conventional imaging, with a particular focus on prospective clinical trials. Searches were conducted through May 21, 2023, with no date restriction. All searches were supplemented by examining reference lists in all relevant publications to identify additional articles for inclusion. The literature search was limited to English-language publications in peer-reviewed journals using the following Medical Subject Headings (MeSH) terms and keywords: ‘prostate neoplasms’; ‘biochemical recurrence’; ‘treatment’. To be eligible for inclusion in this review, the identified records must be reporting or providing recommendations on the risk stratification approaches, salvage treatment options, or lifestyle interventions in adult men with BCR. Database searches yielded 432 articles, of which 95 were included in this review after title/abstract screening and full-text selection.

## Results

### Biochemical recurrence following whole-gland treatment

Traditionally, BCR is defined by a rising serum PSA after primary definitive therapy without conventional imaging (computed tomography and bone scan) evidence of metastasis [17]. However, PSA is not cancer-specific and recurrent benign prostate growth after EBRT or other minimally invasive therapies and, rarely, residual benign prostate tissue remaining in situ post-RP can generate false positives [18, 19]. Therefore, confirmation of BCR prior to treatment is crucial to prevent unnecessary treatment. Despite the existing evidence on the ability of ultrasensitive PSA testing in determining BCR and informing salvage treatment at concentrations ≤0.1 ng/ml post-RP [20–22], the clinical utility of low-detectable PSA values is a matter of debate and the consensus is lacking for the optimal PSA threshold for initiating therapy post-RP; medical societies have proposed several criteria for establishing BCR and monitoring of serum PSA levels after initial definitive therapy (Table 1) [9, 14, 23, 24]. In general, BCR is classified as PSA increases above 0.1 ng/ml post-RP [9, 14]. Post-RP, the failure of PSA to decline to undetectable levels is defined as PSA persistence [9], biologically representing potentially larger residual cancer burden compared with PSA recurrence [25]. Post-EBRT, consensus exists among medical societies that BCR is defined as nadir +2.0 ng/ml [9, 14].

**Table 1:** Treatment guidelines for BCR.

	AUA/ASTRO/SUO [14, 40, 95]		ASCO [8, 24]		EAU/EANM/ESTRO/ESUR/ ISUP/SIOG [16]			NCCN [9]	
	Post-RP	Post-EBRT	Post-RP	Post-EBRT	Post-RP	Post-EBRT		Post-RP	Post-EBRT
BCR definition	PSA increase of 0.2 ng/ml AND confirmatory value of ≥0.2 ng/ml	PSA increase of >2 ng/ml over PSA nadir	Detectable^a^ PSA with a subsequent rise	PSA increase of >2 ng/ml over PSA nadir	PSA > 0.4 ng/ml and rising		PSA increase of >2 ng/ml over PSA nadir	Detectable^a^ PSA that increases on ≥2 confirmatory tests or increases to PSA levels >0.1 ng/ml	PSA increase of >2 ng/ml over PSA nadir
PSA monitoring post-treatment	Yr ≤10: pt preference and risk of recurrence; Yr >10: high-risk patients only^b^		No recommendations		Yr 1: 3, 6, 12 mo; Yr 2–3: every 6 mo; Yr >3: annually			Yr 1–5: 6–12 mo; Yr >5: annually	Yr 1–5: every 6 mo; Yr >5: annually
								High-risk pts: every 3 mo^c^	High-risk pts: every 3 mo^c^
Observation/active surveillance	Observation recommended for pts with BCR and no evidence of metastatic disease by conventional imaging		Active surveillance^d^ can be offered to pts with low-risk BCR post-RP^e^ and/or post-EBRT^f^		Observation recommended for pts with: PSADT > 12 mos AND pathological GS < 8 for RP; interval to biochemical failure >18 mos AND GS < 8 for RT			Observation recommended for pts with no distant metastasis or no prior imaging; as an alternative to EBRT ± ADT	Observation recommended for pts and no distant metastasis; as an alternative to ADT if positive TRUS biopsy and LE ≤ 10 yr; as an alternative to RP + PLND, brachytherapy, cryotherapy or HIFU if positive TRUS and LE > 10 yr
sEBRT (post-RP setting)	Early treatment recommended (PSA levels ≤1.0 ng/ml) with no evidence of distant metastatic disease; ADT + sEBRT recommended when PSA ≥ 0.2 ng/ml		Not recommended		Low-risk BCR, not recommended; high-risk BCR, early treatment recommended (PSA levels ≤0.5 ng/ml)			Early treatment for pts with detectable PSA levels (≥0.2 ng/ml); pts with high Decipher GC scores (GC > 0.6) should be strongly considered for sEBRT and the addition of ADT when early sEBRT is missed	
sADT	Not routinely recommended		iADT may be offered to pts with high-risk BCR after RP^g^ and/or EBRT^h^		Low-risk BCR, not recommended; high-risk BCR (PSADT, <6–12 mo; GS, >7), early treatment recommended			Early treatment for pts with elevated PSA + shorter PSADT ([≤6 mo] or rapid PSA velocity) + LE ≥ 10 yr	
Surgical and non-surgical salvage treatments (post-EBRT setting)	Not recommended		Not recommended		sRP, SBRT, brachytherapy, HIFU, or cryosurgical ablation should only be offered to highly selected pts with biopsy-proven local recurrence as part of a clinical trial or in experienced centers			Cryosurgery and HIFU in the absence of metastatic disease; brachytherapy and sRP for select pts	

*ADT* androgen deprivation therapy, *ASCO* American Society of Clinical Oncology, *ASTRO* American Society for Radiation Oncology, *AUA* American Urologic Association, *BCR* biochemical recurrence, *EAU* European Association of Urology, *EBRT* external beam radiotherapy, *GC* genomic classifier, *ENAM* European Association of Nuclear Medicine, *ESTRO* European Society for Radiotherapy & Oncology, *ESUR* European Society of Urogenital Radiology, *GS* Gleason score, *HIFU* high-intensity focused ultrasound, *iADT* intermittent androgen deprivation therapy, *ISUP* International Society of Urological Pathology, *LE* life expectancy, *mo* month(s), *NCCN* National Comprehensive Cancer Network, *Pt* patient(s), *PLND* pelvic lymph node dissection, *pt* patient, *PSA* prostate-specific antigen, *PSADT* PSA doubling time, *RP* radical prostatectomy, *RT* radiation therapy, *s* salvage, *SIOG* International Society of Geriatric Oncology, *SUO* Society of Urologic Oncology, *TRUS* transrectal ultrasound, *yr* year(s).

^a^There is no consensus of what threshold PSA value is defined as undetectable.

^b^High-risk is defined at initial diagnosis; ≥T3 or GS 8–10 or PSA ≥ 20 ng/ml.

^c^High-risk is defined at initial diagnosis; T3a or GS 8–10 or PSA > 20 ng/ml.

^d^ASCO uses the term active surveillance as opposed to observation in the BCR setting.

^e^Post-RP, low-risk BCR is defined as a PSADT ≥ 1 yr and GS < 8.

^f^Post-EBRT, low-risk BCR is defined as an interval to BCR > 18 mo and GS < 8.

^g^Post-RP, high-risk BCR is defined as a PSADT < 1 yr or GS 8–10.

^h^Post-EBRT, high-risk BCR is defined as an interval to BCR < 18 mo or GS 8–10.

### Risk stratification in patients with BCR

The inherent heterogeneity of BCR presents challenges for optimal disease management, particularly in the context of monitoring treatment response in patients with negative conventional imaging [17]. Given the prognostic value of clinicopathological and genomic parameters in BCR, risk stratification is essential for a personalized approach to the treatment of patients who experience BCR [6, 26, 27]. One of the strongest predictors of metastasis and death is PSADT, a mathematical determination of the time in months required for PSA to increase two-fold in an individual patient [28]. In patients with BCR post-RP, the risk of metastasis and PCSM decrease significantly from the lowest (<3 months) to the highest (≥15 months) PSADT levels [3, 6, 29]. Higher GSs are also strong positive predictors of distant metastatic recurrence, PCSM, and overall mortality in men who develop BCR [5]. In this context, European guidelines suggest that patients with BCR should be stratified by risk of disease progression prior to commencing additional treatment; classifying patients with BCR post-RP as high-risk of disease progression if they have either a PSADT ≤ 1 yr or GS 8–10 and low-risk with a PSADT > 1 yr and GS < 8 [15]. Post-EBRT, patients are defined as high-risk for disease progression if they have either biochemical failure (IBF) ≤ 18 mo or GS 8–10 and defined as low-risk if the interval from primary therapy to IBF > 18 mo and GS < 8 [30]. ASCO and NCCN recommend patients with BCR post-RP and post-EBRT should be stratified by risk of disease progression prior to commencing additional treatment [8, 9]. Based on the findings from a meta-analysis of studies with 44630 patients who underwent either RP or RT [5], the ASCO 2021 guideline update classifies patients with BCR post-RP as high-risk for disease progression if they have either a PSADT ≤ 1 year or a pathologic GS 8–10 (identified on prostatectomy histology report), and low risk if they have both a PSADT > 1 year and a GS < 8 [5, 8]. Post-RT, high-risk BCR is defined as an interval to BCR ≤ 18 months or a clinical GS 8–10 (identified on prostate biopsies), whereas low-risk BCR is defined as an interval to BCR > 18 months and a GS < 8 [5, 8]. Additionally, the NCCN and AUA/ASTRO/SUO guidelines recommend that genomic testing can contribute to development of a patient’s overall risk profile for recurrence [9, 23]. Post-RP, genomic tests that contribute to patient management by assessing expression levels of RNA include Decipher® (Decipher Biosciences Inc., San Diego, CA, USA; 22 genes) [31]. Multivariable analysis of 23 studies (*n* = 12600) demonstrated that the Decipher genomic classifier (GC) score was independently prognostic for biochemical failure, distant metastasis, and PCSM, and improved discrimination of these endpoints over standard of care [32]. In addition, in the G-MINOR multicenter, randomized trial of post-RP patients with PSA < 0.1 ng/ml, a high GC score in the GC arm (*n* = 175) significantly increased the probability of adjuvant treatment (OR 8.8, 95% CI 1.9–39.7, *p* = 0.005) [33].

### Treatment recommendations for patients with BCR after primary definitive therapy

#### Salvage EBRT after RP

Salvage EBRT (sEBRT) is potentially curative and may delay the need for chronic, non-curative treatments, such as long-term androgen deprivation therapy (ADT) [12]. Notably, adjuvant EBRT (aEBRT) and early sEBRT have shown comparable efficacy in prospective trials; however, aEBRT has been associated with a higher rate of adverse events (AEs), particularly acute and late grade ≥2 genitourinary toxicity and grade ≥2 erectile dysfunction [34–36]. Furthermore, retrospective studies have demonstrated that early sEBRT can decrease the risk of all-cause mortality (ACM) and PCSM in patients with BCR [30, 37, 38]. Prognostic factors associated with oncologic outcomes following sEBRT are PSA levels at the time of sEBRT and PSADT [30, 39]. In a retrospective analysis of 5509 men, 1497 of whom experienced BCR (rising PSA ≥ 0.2 ng/ml from two consecutive measurements), early sEBRT (PSA < 0.5 ng/ml) was more effective in reducing the risk of metastatic progression (hazard ratio [HR] 0.32, 95% CI 0.20–0.53; *p* ≤ 0.001) compared with late sEBRT (PSA ≥ 0.5 ng/ml) (HR 0.56, 95% CI 0.35–0.88; *p* = 0.01) [30]. Similarly, the risk of PCSM was significantly lower following early sEBRT (HR 0.31, 95% CI 0.15–0.62; *p* ≤ 0.001) compared with late sEBRT (HR 0.58, 95% CI 0.32–1.04; *p* = 0.07) [30]. Additionally, a prospective institutional real-world study of patients with post-RP BCR from two treatment sites in Germany and the US (1990‒2020) found significant associations between pre-sEBRT PSA cutoffs (>0.10 to ≤0.50 ng/ml) and ACM, reporting a higher 10-year ACM risk estimate with sEBRT at PSA levels >0.25 ng/ml versus ≤0.25 ng/ml (HR 1.49, 95% CI 1.11–2.00; *p* = 0.008) [38].

The European guideline recommends sEBRT for patients classified as high-risk BCR and PSA levels ≤0.5 ng/ml [16]. The AUA/ASTRO/SUO guidelines recommend that patients should be informed that sEBRT for BCR is most effective at PSA levels <1.0 ng/ml [40]. Independent of clinical and pathological risk factors, Decipher risk classification has been shown to factor into the decision-making regarding the timing of treatment intensification for patients with BCR, such as sEBRT [41, 42]. Thus, the NCCN Clinical Practice Guidelines in Oncology (NCCN Guidelines®) recommend patients with high GC scores (>0.6) should be strongly considered for EBRT and addition of ADT when the opportunity for early EBRT has been missed [9].

#### Combination ADT with sEBRT

sEBRT is often combined with ADT for the treatment of men with BCR. Preclinical studies demonstrated that androgen deprivation downregulates vascular endothelial growth factor, leading to apoptosis of endothelial cells and decreased vascularization, and also reduces the dose of EBRT required to control 50% of the tumor, providing a biological basis for combination treatment [43]. First-generation non-steroidal anti-androgens (NSAAs) combined with sEBRT have demonstrated a benefit in men with BCR. In the randomized RTOG 9601 trial of 760 men with BCR post-RP, 24-month treatment with high-dose bicalutamide (150 mg daily) in combination with sEBRT resulted in significantly increased overall survival (OS) rates (*p* = 0.04) [13]. However, improvements in OS were not identified until >10 years of follow-up. Subsequent analyses from this trial showed that the benefit to adding bicalutamide was only observed in patients with a pre-EBRT PSA > 0.6 ng/ml [44]. Indeed, in patients with lower PSA values, bicalutamide had no benefit and actually increased other-cause mortality (*p* = 0.01) [44], further highlighting the importance of risk stratification as well as potential risks of unwarranted treatment intensification. Nonetheless, given the delay until benefits are observed, life expectancy (LE) is an important factor for patients considering hormone therapy [17]. LE can be estimated as the average number of years of life remaining for persons at a certain age using the life table functions developed by the National Vital Statistics System [45]. The life table functions use national data on death and population counts to calculate LE based on the number of survivors and the number of person-years lived at and above a given age [45]. For patients with shorter LE even with severe disease, observation can be the best option. In the GETUG-AFU 16 prospective, phase 3 trial, 6 months of treatment with goserelin plus sEBRT significantly improved 9-year progression-free survival (*p* < 0.001) and metastasis-free survival (MFS; *p* = 0.034) compared with sEBRT alone [46]. Of note, the progression-free survival benefit of combined sEBRT and ADT was observed both in the high-risk (GS 8–10, T3 disease, and/or positive margins) and the low-risk subgroups (*p* < 0.001 and *p* = 0.004, respectively). In contrast, 9-year MFS was comparable between sEBRT+ADT and sEBRT alone for both high-risk and low-risk groups. In addition to consideration of tumor-related factors to determine which patients would benefit from sEBRT in combination with ADT, other patient-related factors that should be considered include comorbidities (frailty, heart disease, osteoporosis, and mental health) and LE [47].

Decipher was used to evaluate tumor samples from 486 patients with recurrent disease collected from RTOG 9601, the randomized trial of sEBRT vs. sEBRT with bicalutamide described above [48]. Adjusted for age, race/ethnicity, GS, T stage, margin status, baseline PSA, and treatment cohort, multivariable analysis demonstrated the GC score (continuous variable, per 0.1 unit) was independently associated with risk of distant metastasis (HR 1.17, 95% CI 1.05–1.32, *p* = 0.006), PCSM (HR 1.39, 95% CI 1.20–1.63, *p* < 0.001), and OS (HR 1.17, 95% CI 1.06–1.29, *p* = 0.002). Furthermore, the 12-year benefit provided by ADT improved OS three-fold in patients with intermediate- and high-risk GC scores compared with low-risk GC scores (8.9% vs. 2.4%). In addition, the patients who received early sEBRT (PSA < 0.7 ng/ml) in combination with ADT with high vs. low GC scores experienced benefits in 12-year risk of developing distant metastasis (11% vs. 0.4%), PCSM (8.4% vs. 1.0%), and OS (4.6% vs. –7.8%). Importantly, patients with low-risk GC scores who would not benefit clinically from treatment intensification could also be identified. Overall, these results demonstrated that genomic profiling may identify patients with BCR, independent of PSA level, who would or would not benefit from sEBRT+ADT combination relative to sEBRT alone.

#### Surgical and non-surgical salvage treatments after definitive EBRT

A number of surgical and non-surgical salvage treatments have been proposed for histologically confirmed localized BCR post-EBRT that include salvage RP (sRP), cryotherapy, high-intensity focused ultrasound (HIFU), stereotactic body radiotherapy (SBRT), and low-dose-rate (LDR) and high-dose-rate (HDR) brachytherapy. Meta-analyses of these treatments demonstrated comparable relapse-free survival rates at 5 years, ranging from <50% with HIFU to approximately 60% with SBRT, cryotherapy, and HDR brachytherapy, while 2-year relapse-free survival rate was significantly lower with HIFU compared with sRP (52% vs. 72%; *p* < 0.001) [49]. The best candidates for re-irradiation with brachytherapy or SBRT have good urinary function and performance status.

In RTOG 0526, LDR brachytherapy re-irradiation was associated with a 14% risk (95% CI 6–21) of late treatment-related grade ≥3 gastrointestinal/genitourinary toxicity [50]. Comparable rates of grade ≥2 genitourinary toxicity resulting from HDR brachytherapy and SBRT have been reported [26, 51–53]. Historically, open sRP has been associated with poor functional outcomes and high complication rates; however, robot-assisted sRP demonstrated reduced adverse outcomes (anastomotic and/or urethral strictures, 16.6% vs. 7.7%, *p* = 0.007) and significant improvements in blood loss and duration of hospital stay (both, *p* < 0.001) compared with open sRP [54]. In patients from both groups who were continent at baseline, urinary continence remained unchanged or improved in 57% of patients, and 24.6% of patients experienced severe incontinence defined as ≥3 pads per day [54]. Thus, European guidelines recommend sRP should only be considered for patients with few comorbidities and LE of ≥10 yrs, pre-sRP PSA < 10 ng/ml with no lymph node involvement or evidence of metastatic disease, and at initial diagnosis, GS ≤ 8 and clinical stage T1 or T2 [16]. NCCN Guidelines® recommend sRP (with pelvic lymph node dissection [LND]) as an option for highly selected patients with local recurrence after EBRT, brachytherapy, or cryotherapy in the absence of metastases [9]. Notably, sRP should be performed in experienced centers or as part of a clinical trial. According to NCCN guidelines, pelvic salvage LND (sLND) can be considered for patients with BCR and pelvic recurrence post-EBRT [9]. Single-center retrospective studies have reported encouraging survival outcomes for sLND in the post-RP node-recurrent setting [55, 56]; however, a retrospective analysis of multi-institutional data did not support the long-term clinical benefits of sLND in patients with MTI-detected post-RP nodal recurrence, with 36% and 34% probability of ACM and PCSM at 10 years, respectively [57]. Cryotherapy and HIFU are other local treatments recommended by European and NCCN guidelines for BCR post-EBRT in the absence of metastasis [9]. LE > 5 years, low or intermediate D’Amico risk category, and low pre-EBRT PSA level are factors associated with improved OS after HIFU [58].

#### Systemic treatment options

Strategies for BCR disease management include first- and second-generation ADT (with or without EBRT) as well as lifestyle interventions. NSAAs competitively inhibit the action of androgens by binding to cytosolic androgen receptors in the target tissue [59]. ADT blocks the release of hormones, such as luteinizing hormone-releasing hormone, and reduces both the rate of testicular androgen synthesis and levels of circulating androgens [60]. Androgen-sensitive PCa responds to treatment that counteracts the effect of androgen and/or removes its source. There is no consensus on the benefit of salvage ADT alone following BCR, therefore, the risk of AEs must be carefully assessed and discussed. A systematic review evaluating the effectiveness of ADT alone for BCR determined that ADT may be appropriate for men with a high risk of disease progression (PSADT < 6–12 months; GS > 7) and long LE [61]. The European and NCCN Guidelines are consistent with the outcomes of this review [9, 16]. The AUA/ASTRO/SUO guidelines suggest that men with high-risk BCR should only be recommended intermittent ADT with no evidence of metastasis [14]. These guidelines also recommend that these men be offered clinical trial enrollment or observation [14]. Despite its potential clinical benefits, ADT is associated with significant AEs, and its long-term use may contribute to an impaired quality of life (QoL), including depression, fatigue, hot flashes, and sexual dysfunction [62]. Long-term ADT also is associated with an increased risk of cardiovascular disease, diabetes mellitus, and osteoporosis [63–66]. Furthermore, ADT can confound PCa tumor imaging and detection [67].

#### Peripheral androgen blockade

NSAAs in combination with 5α-reductase inhibitors, such as finasteride, have been evaluated for delaying ADT initiation in BCR [68–70]. In a study of 37 patients with BCR treated with bicalutamide and finasteride, the median time to progression to ADT was 37.6 months (interquartile range [IQR] 20–75), and from the start of treatment the median time to castration resistance was 49.8 months (IQR 41–not reached). NSAAs plus 5α-reductase inhibitor combinations are not currently recommended in the guidelines, but may be considered for patients who are older and unfit, or carefully selected patients who want to avoid the toxicities of castration therapy [71].

#### Timing of ADT

The TOAD trial investigated the impact of delayed vs. immediate ADT treatment on OS in 293 men, of whom 261 experienced PSA relapse after curative therapy [72]. The 5-year OS was modestly increased in the immediate therapy arm vs. the delayed therapy arm (91% vs. 86%, *p* = 0.047). The study also found that immediate therapy was associated with a lower incidence of local progression (13% vs. 20%) and a significantly longer time to local progression (adjusted HR 0.51; 95% CI 0.34–0.76; *p* = 0.001), compared with delayed therapy. Nonetheless, time to distant progression was not significantly different between the immediate and delayed therapy 1-year or 6-year follow-ups. Immediate therapy also was associated with minimal diminishment in QoL; however, an increased percentage of patients experienced serious AEs vs. delayed therapy (41% vs. 32%). Moreover, despite the stratification by PSADT, the study did not report the subgroup analysis on BCR-experiencing patients with shorter (<10 months) vs. longer (≥10 months) PSADT, limiting the application of these findings to the management of high-risk BCR. As the evidence published thus far only demonstrates a modest clinical benefit in OS for early ADT and that side effects associated with chronic ADT therapy are an important consideration, only high-risk patients should consider early ADT according to both NCCN and European guidelines (defined by shorter PSADT and long LE) [9, 16].

#### Intermittent ADT

Intermittent ADT (iADT) has been proposed as an option that may delay disease progression while providing relief from the AEs and complications associated with continuous dosing [11, 73]. The Canadian PR.7 study demonstrated the non-inferiority of iADT compared with continuous ADT (cADT) with respect to OS in patients (*n* = 1386) with BCR post-EBRT (≥3 ng/ml increase over nadir PSA) together with improved QoL improvements in the iADT cohort [11]. Due to a high number of deaths (59%) unrelated to PCa, a *post hoc* analysis of PCa-specific survival was also conducted showing non-comparable deaths from PCa or related causes in the iADT (*n* = 120) vs. the cADT (*n* = 94) group (*p* = 0.13) [11]. A meta-analysis of 15 clinical trials, representing 6856 men with PCa who underwent iADT or cADT, concluded that certain physical and sexual functions improved with iADT, but there were no major between-group differences in OS, time to castration resistance, QoL, or AEs despite a lower trend in point estimates for iADT [74]. For patients with nonmetastatic BCR, the NCCN Guidelines recommend iADT, with no specific recommendations for patient selection [9]. Alternatively, a consensus statement reached by an expert panel of US-based uro-oncologists recommended IADT should be only considered for high-risk patients (defined as PSADT ≤ 9 months and GS ≥ 8) with early BCR (<3 years); low- and intermediate-risk patients with BCR should undergo observation [73]. Consistent with this, the ASCO guidelines recommend that iADT may be offered to patients with high-risk BCR after RP (PSADT ≤ 1 year or a pathologic GS 8–10) or RT (interval to BCR ≤ 18 months or a clinical GS 8–10); active surveillance may be considered in those with low-risk BCR after RP (PSADT > 1 year and GS < 8) or RT (interval to BCR > 18 months and GS < 8) [8].

#### Second-generation androgen-targeted therapies

Second-generation antiandrogen therapies have been developed with increased androgen receptor specificity and affinity, compared with their NSAA predecessors [75]. Enzalutamide, the first characterized second-generation NSAA, improved OS in both nonmetastatic castration-resistant and metastatic castration-sensitive PCa [76, 77]. Notably, in the STAMPEDE trial (NCT00268476), patients with high-risk nonmetastatic castration-sensitive PCa (nmCSPC) who received abiraterone acetate plus prednisolone with or without enzalutamide plus ADT for 2 years demonstrated significantly improved MFS and OS (both *p* < 0.001), compared with ADT alone [78]. The further addition of enzalutamide did not impact efficacy outcomes, but increased grade ≥3 AEs (57% vs. 37%). In contrast, the global phase 3 EMBARK trial (NCT02319837) demonstrated significant and clinically meaningful improvements in MFS for patients with high-risk BCR (PSADT ≤ 9 months) and negative conventional imaging who received enzalutamide plus leuprolide (HR 0.42, 95% CI 0.30–0.61; *p* < 0.0001) or enzalutamide monotherapy (HR 0.63, 95% CI 0.46–0.87; *p* = 0.005) vs. placebo combined with leuprolide after a median follow-up of 60.7 months [79]. A summary of other ongoing clinical trials in patients with BCR is presented in Table 2.

**Table 2:** Active clinical trials evaluating pharmacotherapy at BCR after primary treatment.

Study phase	Therapy	Estimated (actual) enrollment	Comparator	Primary endpoint	Estimated completion date	NCT
2	Apalutamide + ADT + sEBRT, docetaxel	40	None	PFS at 36 mo	Completed	NCT03311555
2	RV001V	180	Placebo	Time to PSA progression (up to 3 yr)	November 2022	NCT04114825
2	Enzalutamide + sEBRT	96	sEBRT + placebo	Freedom from PSA progression (assessed 2 yr from end of therapy)	December 2023	NCT02203695
3	Apalutamide + LHRH analog, apalutamide, abiraterone + prednisone, LHRH analog	504	LHRH analog (degarelix or leuprolide + bicalutamide)	PSA PFS up to 36 mo	January 2024	NCT03009981
2	Olaparib, durvalumab	32(5)^a^	None	Number of participants with an undetectable PSA (assessed after 1 yr)	February 2024	NCT03810105
	Olaparib	50^b^	None	Response rate as measured by 50% decline in PSA from baseline (assessed after 4 wk)	February 2024	NCT03047135
2	pTVG-HP, nivolumab, GM-CSF	41(19)	None	Percentage of pts within acceptable toxicity boundaries (up to 48 wk); PSA CR rate (up to 48 wk)	January 2025	NCT03600350
2	GnRH + bicalutamide + sEBRT, GnRH + abiraterone + apalutamide + prednisone + sEBRT	345	None	PSA PFS at 2 yr	December 2025	NCT03141671
2	Nivolumab	15^c^	None	Percentage of pts with a ≥ 50% decline in PSA (up to 6 mo post-intervention)	January 2025	NCT04019964
2	Nivolumab	34	None	Proportion of pts with high-risk BCR that experiences PSA decline or stabilization after 12 wk of treatment	March 2025	NCT03637543
2	Pactritinib	46	None	Number of pts with 6-mo PSA PFS	June 2026	NCT04635059
2	sEBRT + ADT for 24 mo (triptorelin)	394	sEBRT + ADT for 6 mo (triptorelin)	MFS at 5 yr	February 2029	NCT04242017
2	Enhanced ADT (enzalutamide + GnRH analog) + EBRT	242	Standard ADT (GnRH analog ± bicalutamide) + sEBRT	PFS (assessed up to 5 yr)	September 2029	NCT03809000
3	Apalutamide + LHRH + EBRT	490	LHRH + EBRT	PFS at 5 yr	December 2033	NCT04181203

*ADT* androgen deprivation therapy, *BCR* biochemical recurrence, *CR* complete response, *EBRT* external beam radiation therapy, *GM-CSF* granulocyte-macrophage colony-stimulating factor, *GnRH* gonadotropin-releasing hormone, *LHRH* luteinizing hormone-releasing hormone, *MFS* metastasis-free survival, *NCT* national clinical trial number, *PFS* progression-free survival, *pt* patient, *PSA* prostate-specific antigen, *s* salvage, *wk* week(s), *yr* year(s).

^a^BCR is defined as rising PSA (50% increase to 0.5 ng/ml or more based on at least three determinations obtained 1 wk apart).

^b^BCR is defined as PSADT of ≤6 mo and minimum PSA of ≥1 ng/ml.

^c^Absolute PSA ≥ 1.0 ng/ml at screening.

### Other treatment options

#### Lifestyle

Dietary polyphenols, such as curcumin, have demonstrated inhibition of PCa growth in preclinical models and may complement a treatment or prevention strategy in men with PCa [80]. In a randomized controlled trial, patients with BCR after localized treatment or metastases at diagnosis were given iADT [80]. At ADT discontinuation, men were randomized (1:1) and received either curcuminoid powder capsules (1440 mg/day for 6 months) or placebo. While there was no significant difference in ADT “off-treatment” time, curcumin treatment significantly lowered PSA progression rate compared with control (10% vs. 30%; *p* = 0.026) [80]. A phase 2, single-arm study found that polyphenol-rich pomegranate juice prolonged PSADT, compared with baseline [81]; however, subsequent randomized trials found no differences in on-study PSADT between low- and high-dose pomegranate extract and between pomegranate extract and placebo [82, 83]. As such, these data do not support the use of pomegranate juice/extract for patients with BCR. Whole food supplements containing polyphenols have also demonstrated significant improvements in PSA levels for patients with BCR [84]. Patients with BCR (*n* = 199) randomized to a polyphenol-rich whole food supplement containing pomegranate, green tea, broccoli, and turmeric or placebo for 6 months demonstrated significant differences in the percentage increase in PSA (15% vs. 79%; *p* < 0.001) and percentage of patients with stable or lower PSA at the end of the study (46% vs. 14%; *p* < 0.001) [84].

Lifestyle interventions, such as weight loss and low-carbohydrate (LCD) and low-fat diets, have been studied in patients with BCR without affecting PSA or PSADT [85, 86]. A study of 57 patients with BCR randomized to LCD (*n* = 30) or control (*n* = 27) demonstrated that an LCD over 6 months did not significantly impact PSADT (*p* = 0.31) [86]. However, a *post hoc* analysis adjusting for key baseline covariates, including baseline PSA, pre-study PSADT, and prior treatment, in addition to hemoconcentration during the study, found that PSADT was significantly lowered in the LCD group (*p* = 0.007) [86]. Larger prospective studies are warranted to evaluate the impact of LCD on PCa disease progression. An evaluation of all the various dietary and lifestyle changes employed to manage PCa is beyond the scope of this paper; however, this topic has been reviewed elsewhere [87, 88].

## Discussion

**Fig. 1:**
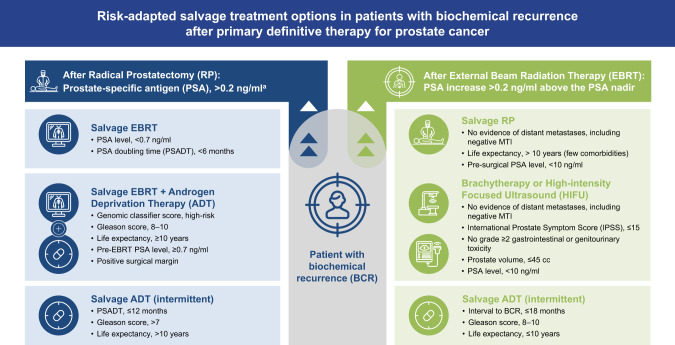
Risk-adapted salvage treatment options in patients with biochemical recurrence after primary definitive therapy for prostate cancer. Clinicopathological and genetic factors recommended by medical societies and expert groups for the consideration of salvage treatments in patients with BCR [8, 9, 16, 48, 94].

The treatment landscape of PCa has dramatically changed due to rapid therapeutic advancements, including MTI, genomic testing, and novel agents. While these developments are helpful, level-one evidence to guide clinicians prescribing treatment for BCR is lacking; thus clinical factors, such as PSADT, GS, and genomic testing can be applied to estimate the risk for PCa progression [73, 89]. A short PSADT (<9 months) is associated with increased risk of clinical progression, metastasis, and PCSM [3]. However, patients with BCR may require salvage treatment, and the decision-making must balance risk–benefit assessment (Fig. 1). Besides post-RP sEBRT, there are limited standard treatment options for men with BCR, and identifying optimal therapy remains an unmet need [9, 14]. Post-EBRT, salvage local therapies recommended for select patients include surgical and non-surgical options that have demonstrated similar relapse-free survival rates but differing AE profiles. For patients who have exhausted local treatment options, the AUA/ASTRO/SUO guidelines promote observation and clinical trial enrollment [14]. They do not recommend ADT and advise that, if used, it should be employed intermittently due to modest QoL improvements in patients with BCR. Second-generation anti-androgens have increased androgen specificity and affinity compared with their predecessors, with emerging phase 3 trial data demonstrating improved MFS following enzalutamide treatment in patients with high-risk BCR [76, 79, 90].

MTI will change the management of BCR and, in the future, may be as much of a stratification factor as PSADT, grade score, or genomic and molecular profiling. The results from three studies indicate that application of MTI to identify patients with BCR that would benefit from treatment may have a significant impact on patient outcomes, for example, the consideration of metastasis-directed therapy, thus specifically delaying the commencement of ADT [91–93]. The ultimate goal of treating BCR is to improve clinical outcomes with delayed disease progression and prolonged OS while minimizing AEs and preserving QoL. Thus, definition of BCR needs to evolve to match the increased sensitivity of PSA assays and MTI in detecting recurrence/metastasis at PSA levels below the traditional cutoffs.

In conclusion, despite a current lack of consensus for BCR treatment among guideline associations and medical societies, stratification of patients by risk is essential, assessing the potential AEs and clinical benefits of therapeutic strategies. According to the ASCO guidelines, active surveillance can be considered in low-risk BCR, whereas in higher-risk disease, iADT may be appropriate. European guidelines, AUA/ASTRO/SUO and NCCN recommend observation for select patients with BCR and no evidence of distant metastasis after RP or EBRT. Cryotherapy, HIFU, and, in selected patients, brachytherapy and sRP are local treatment options recommended for these patients by the European guidelines and NCCN. The AUA/ASTRO/SUO guidelines recommend early sEBRT for BCR post-RP, with the addition of ADT when early treatment is missed. The European guidelines and NCCN only recommend sEBRT for patients with BCR post-RP who have high-risk features. Additionally, early salvage ADT can be considered for higher risk patients with BCR and a long LE. Nevertheless, clinical data on the optimal treatment of patients with high-risk BCR after primary PCa treatment are limited. Results from ongoing clinical trials will address this unmet medical need and may provide additional treatment guidance.
